# Engagement of Families Attending Early Childhood Services During 5-Month School Closure Due to COVID-19: An Italian Experience

**DOI:** 10.3389/fpsyg.2021.722834

**Published:** 2021-11-18

**Authors:** Roberta Nossa, Emilia Biffi, Giovanna Colnago, Giovanna De Gregorio, Laura Saudelli, Gianluigi Reni, Christian Caruso

**Affiliations:** ^1^Scientific Institute, IRCCS Eugenio Medea, Bosisio Parini, Italy; ^2^Research & Development Department, Cooperativa Sociale Aeris, Vimercate, Italy

**Keywords:** COVID-19, school closure, childhood services, family engagement, child development, child well-being, children, parents

## Abstract

During the COVID-19 outbreak, we experienced the suspension of both work-related and spare activities, with the closure of shops, companies, services, as well as schools. Children probably are the ones who have suffered the most from this situation, due to the limited socialization with peers and boredom experienced at home. In this context, schools and childhood services tried to relieve the negative effects brought by the pandemic through actions aimed at actively engaging students and their parents in promoting child development and wellbeing. Therefore, several worldwide actions have been implemented to guarantee educational continuity. However, most of these actions targeted 3–18years old children/adolescents, while the subgroup 0–3 was rarely included. *Cooperativa Sociale Aeris*, a social enterprise based in northern Italy that deals with socio-educational and welfare services, took several measures to overcome problems resulting from the closure of its services dedicated to 0–3 aged children. In this manuscript, we depict how Aeris kept engaged children and their parents, reporting families’ evaluation on the actions taken. For assessing their proposed activities, Aeris promptly distributed an on-line survey to the families in May 2020. The answers showed that the organized activities had a positive impact on both children and parents, diminishing the sense of loneliness and boredom for the former, and acting as an important support for the latter. Therefore, this manuscript could work as a reference for policy-makers and managers of educational services in implementing activities and initiatives during home schooling.

## Introduction

Early childhood education holds a key place in the wellbeing of families and their local communities. These services give opportunities for children’s development and socialization and for enabling families to engage fully in the labor market, each of which is important in contributing to stronger families (Baxter, 2015). Early Childhood Education and Care (ECEC) policies and practices vary among different countries and communities (Rutanen et al., 2014), but they are all based on the universal ideals of the “best interest of the child” (Convention on the Rights of the Child, 1989). The experiences of the first 3years of life are recognized as highly important with respect to child well-being and development: therefore, from the basic care of younger children under age 3 while parents work, the role of Early Childhood Services has been shifted toward socialization, development, and cognitive stimulation (Kamerman, 2000). This role may be made difficult by events that require school closure and children confinement.

The COVID-19 pandemic has drastically changed our lives, leading to the suspension of many activities that were part of our routine. Children are probably the ones who suffered the most from this situation due to the limited social connection, reduced physical activity, loneliness, and boredom that experienced during the COVID-19 outbreak. This could result in long-term negative effects, since the mental and physical health, as well as productivity in adult life, are deeply rooted in the childhood years (Pedrosa et al., 2020).

In this situation, childhood services and schools had to bridge the gap between need for children to learn and socialize and their isolation due to the pandemic, through actions aimed at actively engaging families in promoting child development and wellbeing. For this reason, following the COVID-19 outbreak, schools all over the world reorganized and activated new services to support families and guarantee children educational continuity (see for example, Bubb and Jones, 2020; Caffo et al., 2020; Dong et al., 2020; Dube, 2020; Ferraro et al., 2020; Parmigiani et al., 2020; Putri et al., 2020; Rasmitadila et al., 2020; Zhao et al., 2020; Harper et al., 2021). However, these studies mainly included children and adolescents from 3years old up to 18years old, and only few studies included children younger than 3years and their families (Listyaningrum et al., 2020; Szente, 2020; Lee et al., 2021; Meoded Karabanov et al., 2021).

Being one of the first countries that had to deal with the COVID-19 pandemic, Italy promptly forced a lockdown with the closure of all services among which the schools. The partial school closure is registered the 24th of February 2020, while the total closure the 10th of March 2020, placing Italy as the first European country and the third country in the world after China and Mongolia to close schools (UNESCO Website, 2021). Therefore, Italy above every other European country had to timely respond to the sanitary emergency, organizing actions aimed at facing problems arising from the closure of childhood services and schools that lasted till the end of August 2020. Each school activated custom solutions to be in contact with families, including e-learning and online meetings (Lucisano, 2020; Thorell et al., 2021). The Italian Educational Research Society (SIRD) distributed all over Italy an online survey to investigate the experience of Italian teachers during the COVID-19 outbreak (Lucisano, 2020). SIRD found that different means were used for maintaining contacts with families (e.g., social media, online platforms, websites, blogs, emails, apps etc.) and several didactic strategies were implemented (e.g., online lessons, registered lessons, presentations from students etc.). However, the survey was distributed to teachers from preschools, elementary schools, and secondary schools, excluding the nurseries from this evaluation. To our knowledge, there is a lack of Italian studies specifically dedicated to 0–3 aged children, which is consistent with worldwide findings previously mentioned. In Italy, this is probably due to the lower nursery attendance with respect to schools for 3–18 aged children/adolescents. Indeed, in 2019 only 7% of children under the year attended the nursery, rising to 51% if considering children of 24–36months. On the other side, more than 90% of 3–5 aged children attended the pre-school and the totality of children/adolescents the Italian compulsory school (6–16years; ISTAT, 2020). Moreover, we have to consider the difficulties in organizing online activities for children less than 3years old, since their attention to a screen is easily lost, especially if considering the youngest (<1year old) (Szente, 2020).

In this context, *Cooperativa Sociale Aeris* (hereinafter referred to “Aeris”), a social enterprise based in northern Italy that deals with socio-educational and welfare services, took several measures to overcome problems resulting from the closure of its Early Childhood Services dedicated to 0–3 aged children. Since the beginning of the pandemic, Aeris team found new ways of caring for children and elaborated a specific plan to maintain contact with families, in order to cultivate relationships, oversee situations of greater fragility, and offer support to families.

Therefore, the aim of this manuscript is to depict how AERIS kept engaged children and their families attending Early Childhood Services, and how families evaluated the actions done to involve them and to support child development and wellbeing.

## Materials and Methods

### The Aeris Cooperative: Offered Services and Their Participants

**Figure 1:**
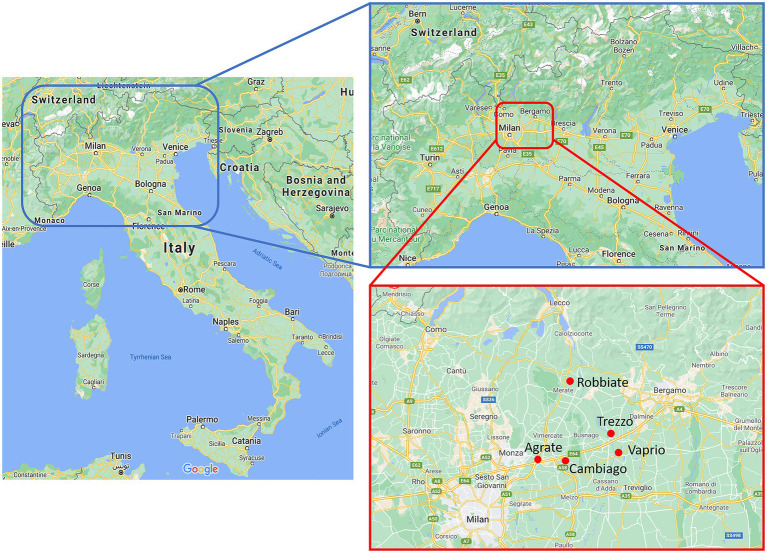
Location in Northern Italy of the Early Childhood Services located in Agrate, Cambiago, Vaprio, Trezzo, and Robbiate.

Aeris is a social enterprise that provides educational and social assistance services, aimed at families with their infants, toddlers and children, youngsters, people with disability, adults in situations of fragility, asylum seekers, and refugees. One of their activities deals with the management of the following Early Childhood Services located in the Northern Italy (precisely in Agrate, Cambiago, Robbiate, Vaprio, and Trezzo, as shown in Figure 1), which are dedicated to 0–3 aged children:

- Four nurseries located in Agrate, Cambiago, Robbiate, and Vaprio, i.e., educational and social services that, in collaboration with families, favors the harmonious development of the child’s personality, promoting his/her independence and socialization. Before the pandemic, most of the time spent at the nursery was dedicated to the “care” of the child (i.e., feeding, washing, changing, putting the child to sleep, cuddling, and consoling).
- Four *“Spazi Gioco”* (SG hereafter) located in Agrate, Cambiago, Trezzo, and Vaprio, i.e., places where the child and his/her caregiver interact within these social spaces. For children who do not attend the nursery, the SG is an opportunity to meet other children, offering stimuli and opportunities for experimentation. In addition, a pedagogist is present to accompany the caregivers in comparisons and reflections on issues relating to their children’s growth. Finally, formative/informative meetings dedicated to families are organized and conducted by different professional figures (i.e., pediatricians, pedagogists, and psychologists).
- “*Merenda in Gioco”* (MG hereafter) in Agrate, a service that gives children the possibility to have a snack and play with educators two mornings a week. MG offers children a space to meet and socialize, with interesting and varied experiential activities aimed at developing expressive, social, communicative, motor, and cognitive skills.
- “*Servizio Ponte”* (SP hereafter) in Agrate, an integration service between nursery (0–3years) and preschool (3–6years), which offers a bridge class co-managed by nursery educators and teachers. Here children meet and socialize, with interesting and varied experiential activities aimed at developing expressive, social, communicative, motor, and cognitive skills, as well as experiencing distance from their parents.

These services have been attended in the academic year 2019–2020 by 228 families distributed as follows: 60 at the Agrate nursery, 24 at Cambiago nursery, 32 at Robbiate nursery, 40 at Vaprio nursery, 30 at Agrate SG, eight at Vaprio SG, 18 at Trezzo SG, and 16 at Cambiago SG. Families enrolled at MG and SP were the same attending the Agrate SG.

However, due to the social distancing imposed during the COVID-19 pandemic, a reorganization of these activities through remote actions became mandatory. This reorganization involved the development of several activities that could be enjoyed by means of technological devices as described in the following section.

### Aeris Proposed Activities During the COVID-19 Outbreak

After the school closure occurred the 24th of February 2020, the Aeris team started to contact families giving information about the re-organization of their services. From the 10th of March, systematic online activities were organized to keep contacts with families, due to the new restrictions following the Decree of the 9th of March 2020 released by the Italian Ministry of Health. The online educational activities lasted until the end of June 2020, while in July, the face-to-face activities restarted in the form of summer camps. During the closure period, the Aeris team had to develop several remote activities, using the guidelines given by the Italian government about the distance educational connections (*Legami Educativi A Distanza*, LEAD; MIUR, 2020). The Smart-Edu website^1^ was created, whose objective was to guarantee and maintain relational and educational continuity with children and families posting stories, videos, tutorials, and chores. It was freely accessible by means of computers, tablets, and smartphones and continuously updated 2/3 times per week to keep contacts with families despite the distance.

In addition to the Smart-Edu website, Aeris’ members used different media to maintain and maximize contacts with families, sharing materials, and information through:

- WhatsApp groups/broadcasts: families received communications *via* WhatsApp approximately three times a week.
- Facebook pages (where posts were published 3/4 times per week):
  - *Aeris 0–3 insieme a piccoli passi*:^2^ the page specifically dedicated to the nurseries;
  - *Spaziogioco papaveriepapere*:^3^ the page specifically dedicated to the SG;
  - *Smart-Edu*:^4^ the page associated with the Smart-Edu Website and activated from 4 March 2020.
- YouTube channel: www.youtube.com/c/AerisCooperativaSociale
- Emails;
- Video calls (*Via* Google Meet or Zoom).

Finally, Aeris concretely moved in two directions to support parents and children:

- It engaged parents with several activities, allowing them to actively intervene in supporting child development during the COVID-19 outbreak, and it provided support especially to the most fragile realities. These activities are presented in detail in the section “Actions to support the parents.”
- It organized activities specifically dedicated to children in order to mitigate the hardship caused by the social distancing imposed during the lockdown. These activities are presented in detail in the section “Actions to support the children.”

### Actions to Support the Parents

Interaction between families and childhood services is at the basis of a correct child development, independently from the educational setting. During the COVID-19 pandemic, the communication was intensified to monitor and support families, especially those more fragile, with the intent not to leave anyone alone. Therefore, Aeris offered the following possibilities:

- Being included in WhatsApp groups or broadcasts to have faster and immediate communication.
- Attending calls and video calls with the educators, having the possibility to attend both group and “one-to-one” calls. Group meetings allowed parents to compare and share difficulties with other families and the Aeris team; one-to-one calls were dedicated to support families in facing specific situations and needs.
- Attending pedagogical consulting by calls or video calls.
- Writing and discussing by email with the educational team. Moreover, Aeris sent bi-weekly emails to update families about the closure/suspension of the services and implemented measures to contain the contagion.

WhatsApp groups/broadcast, emails, the Smart-Edu website, and social pages were used to share with parents written documents and videos dealing with children’s growth, but also documents and videos on current pedagogical topics of interest that could help families in facing this difficult period.

### Actions to Support the Children

During the closure of the Early Childhood Services, the educational teams shared materials and contents every other day. The activities were prepared considering children developmental areas (e.g., gross- and fine-motor development, language, and autonomy) as described in the pedagogical guidelines defined by the Italian Ministry of Education, which are inspired by developmentally appropriate practices (MIUR, 2020). This material was focused on:

- Videos with Italian and English songs and nursery-rhymes accompanied by gestures and musical instruments.
- Videos with stories and readings proposed as at the nursery.
- Video tutorials that explained to parents how to realize simple activities that could be done at home with easily available materials.
- Videos providing ideas and suggestions to parents for engaging children in daily life, such as participating in household chores and taking care of themselves.
- Tutorials that explained to parents how to realize manipulative and graphic workshops, science experiments, sensory explorations, hand-eye coordination activities, and motor games.

In addition, calls and video calls that involved both children and parents replicating the typical routine moments of the nursery were organized once or twice a week, dividing families in small groups (i.e., maximum 15 children).

### Evaluation of the Proposed Activities

With the aim of assessing if the adopted organization and proposed activities had positive feedback on families; a survey was developed by means of Google forms. It was distributed in May 2020 to the 228 families attending the Early Childhood Services. A five-point Likert scale ranging from 1 (strongly disagree/not at all satisfied) to 5 (strongly agree/extremely satisfied) was used to evaluate families’ engagement and satisfaction. Some open-ended questions were also included, allowing families to express their personal feelings and experiences related to the proposed activities. The survey template is presented in the Supplementary Material.

### Statistical Analysis

Median values were computed for each answer given to questions scored with the five-point Likert scale. They are reported as median values (IQR), where IQR is the interquartile range (i.e., the difference between the 3rd and 1st quartiles). A chi-square test was carried out on the results to verify if frequencies of categorical data were uniformly distributed. Finally, *post hoc* power analysis was performed. The statistical significance was established at *p*<0.05.

## Results

Two hundred and twenty-eight families had access to the survey and 119 actively responded (81% were Italian). The majority of them were aged 31–40years (58.5%) and 41–50years (30.7%), few were aged 20–30years (9.4%), and very few were older than 51years (1.4%). The children (aged 0–3years, precisely 26.4±8.1months) were distributed among the Early Childhood Services as follows: 33.6% attended the Agrate nursery, 12.6% Cambiago nursery, 12.6% Robbiate nursery, 12.6% Vaprio nursery, 10.9% Agrate SG, 7.6% Trezzo SG, 6.7% Vaprio SG, 2.5% Cambiago SG, 4.2% Agrate MG, and 6.7% Agrate SP. Among them, 3.4% attended more than one Early Childhood Service but they answered once.

The survey showed that all families have a device with internet connection. Most of them used smartphones (80.7%), but also computers (55.5%), tablets (26.8%), and smart TVs (3.3%). However, not all families have an unlimited internet connection, and 21.8% of them cannot use the service without limits. Moreover, 52.1% of the families have more than one child (aged 0–15years), and 31.1% have at least two children who use devices for online activities.

Most of the families (89.1%) participated to the activities suggested and the 62.2% considered useful to receive feedbacks on other families and educators’ activities by means of WhatsApp (44.6%), email (16.2%), video call (9.5%), Facebook (5.4%), YouTube (4.1%), and Smart-Edu website (4.1%). In particular, the success of social media in sharing the material during the lockdown is demonstrated by the increased number of followers and likes of the Facebook pages, as reported in Table 1.

**Table 1:** Statistic numbers of the Facebook pages managed by Aeris.

Facebook page	Followers’ number in March 2020	Followers’ number in June 2021	Number of likes in March 2020	Number of likes in June 2021
*Aeris 0–3 insieme a piccoli passi*	240	946	233	899
*Spaziogioco papaveriepapere*	251	558	245	538
*Smart-Edu*	0	1,170	0	1,131

*The initial null number of followers and likes referred to the Smart-Edu page is related to the fact that the page was appositely created during the COVID-19, while the other pages were already active*.

Table 2 shows the proposed means to maintain the connection between the Early Childhood Services and families, reporting also the percentage of families who effectively used these means, their satisfaction indexes (SI, expressed as the median score of the five-point Likert scale) with the IQR and the results of the chi-square test.

**Table 2:** Proposed means to maintain contacts between the Early Childhood Services and families.

Proposed means to maintain families/services connection	Families engagement	SI (IQR)	Chi^2^	*p*
WhatsApp groups and broadcasts to shear materials and information	89.9%	4 (1)	90.7	<0.0001
Written documents dealing with children growth	71.4%	4 (1)	93.6	<0.0001
Calls and video calls between the educators and parents (one-to-one)	61.3%	5 (1)	117.1	<0.0001
Smart-Edu website	58.8%	4 (1)	60.6	<0.0001
Video calls among families and educators (group calls)	57.1%	5 (1)	79.5	<0.0001
Videos dealing with children growth	54.6%	4 (1)	75.3	<0.0001
Calls and video calls between the pedagogists and parents (one-to-one)	13.4%	4 (2)	13.2	0.01

*SI, satisfaction indexes; IQR, interquartile range; and Chi^2^, chi-square test. The table also shows the percentage of engaged families, the SI with the IQR (interquartile range), and the results of the chi-square test (p<0.05)*.

As shown in Table 2, most of the families used the proposed means to maintain contacts and appreciated them, as demonstrated by the high satisfaction indexes (SI≥4). Only the calls with the pedagogist did not engage the families as expected (family engagement <50%), even if those who participated appreciated them (SI≥4). Results of the chi-square test (chi^2^ ranging between 13.2 and 117.1, *p*≤0.01) show that SI distributions in Table 2 are non-uniformly distributed in a statistically significant way, i.e., the satisfaction level is strongly unbalanced toward high scores.

**Figure 2:**
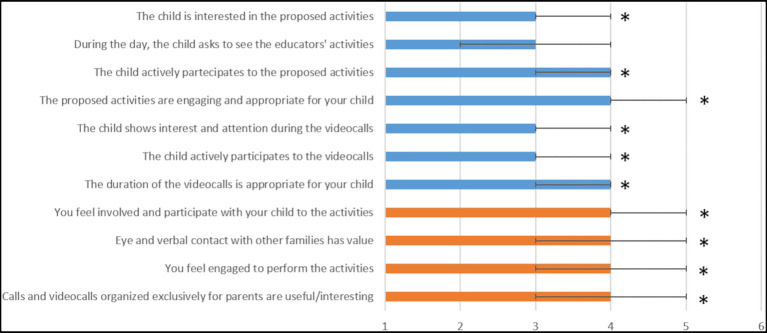
Interests in children (as reported by parents) and parents regarding the proposed activities. Blue bars refer to the children’s involvement, orange bars to the parents. ^*^Chi-square test, *p*<0.0001.

Figure 2 analyzes children (as reported by parents) and parents’ interest in the proposed activities, reporting the median score of the five-point scale and its IQR; blue bars refer to the children’s involvement, while orange ones refer to the parents’.

Both parents and children positively welcomed the proposed activities, judging them interesting as demonstrated by the median score always ≥3. Also in this case, comparisons of score distributions with an equidistributed matrix reached statistical significance for all five-level variables (chi^2^ ranging between 25.7 and 101.7, *p*<0.0001); the only exception regards the item “During the day, the child asks to see the educators’ activities,” whereby no statistical significance was found (*χ*^2^=5.7, *p*=0.23). This could be anticipated due to the age of the children (32% of them were ≤24months old). However, among children older than 24months, 73% of them asked to see the educators’ activities (median score ≥3). To validate these findings, we performed a *post hoc* power analysis on 119 families considering the two main outcomes: children and parents’ engagement. For both the cases, the power of results reached 99.9%.

In addition to these considerations, from the open-ended questions it emerged to send more rhythmic and musical games, dances, and psychomotor activities, and to continue to send readings and song videos (preferably with educators’ voices and faces to maintain the contact), as well as crafts to be done with materials that can be easily found at home. With specific attention to the group video calls, families found them useful, but sometimes chaotic and dispersive. Therefore, they suggested limiting the number of participants, the free discussion, and slowing down the pace during the video calls, also organizing more than one meeting per week.

## Discussion

Due to the COVID-19 outbreak and the suspension of the educational activities, February and March 2020 were accompanied by a general unpreparedness and uncertainty, and the need for everyone (both the educational teams and families) to adapt in a few days to a situation that no one had ever experienced before. To deal with this new destabilizing reality, Aeris activated an early intervention and developed some activities aimed at maintaining contact with families and children, supporting the most critical realities. Aeris moved from the beginning of the pandemic to minimize children’s detachment from their routine, allowing them to experience the lockdown as an active process. With this purpose, the Smart-Edu website was developed; here families and children found chores, stories, videos, e-learning activities, and several resources to decrease the sense of isolation and facilitate long-distance relationships. Aeris also enhanced social channels such as YouTube and Facebook, and created WhatsApp groups/broadcasts with the aim of having contacts with families despite the distance. In particular, sharing material *via* social channels allowed reaching families not enrolled in the Aeris services, as demonstrated by the increased number of followers during the pandemic, leading to the spread of these support initiatives. Lot of importance was given to the video calls, which allowed children to keep having a visual contact with the educators, and parents to share experiences, opinions, and ideas with the educators, pedagogues, and other families.

The activities proposed by Aeris were defined considering the children developmental areas described in the pedagogical guidelines defined by the Italian Ministry of Education, which are inspired by developmentally appropriate practices. These guidelines suggest to foster the following areas of development: gross-motor development, fine-motor development, language, physical wellbeing and autonomy, development of the creative expression, attention and cognitive development, social-emotional development, exploration and play, problem solving, development of the mathematical and scientific thinking, and development of the learning approaches (MIUR, 2020). For each of these areas, the child has to achieve specific skills, and the activities were designed in order to stimulate or acquire these skills. For example, songs and nursery-rhymes accompanied by gestures helped in the development of the language and gross-motor functions, while manipulative and graphic workshops in the development of the fine-motor functions.

In parallel to these actions aimed at guaranteeing the appropriate child’s development, other interventions are necessary to support caregivers in their interaction with children, both for in presence or virtual learning. Specifically, individualized and targeted meetings between families and institutional caregivers are necessary to define the pedagogic intervention on the child and help the educators to define the child’s profile and share it with the parents. A continuous exchange between families and educators is another key-point: the mutual support between families and institutional caregivers facilitates a correct interaction with children and, therefore, the development of their skills. The activities proposed by AERIS to families during school closure aimed also to reinforce the relationship between families and Early Childhood Services thus facilitating the interaction with children.

With the aim of assessing the proposed activities, an on-line survey was distributed to the families in May 2020. The results showed that these initiatives had a positive impact on children and parents, diminishing the sense of loneliness and boredom for the former, and acting as an important support for the latter. Readings, songs, dances, and rhythmic activities were evaluated as the most engaging. The videos proposed by Aeris were so successful that some families decided to propose their own videos and share them with other families. This allowed the families both to fill the void left by this pandemic and to be a support for other families too. Because of these actions, a deeper connection has been created with both other families and the Aeris team: if before the pandemic meetings were organized in more formal contexts, during these months everyone “entered” into others’ homes, both symbolically giving a support, but also more practically through the video calls that allowed seeing others in their home context. Video calls were judged an important tool to reduce social distancing and were widely used, but sometimes they resulted chaotic and dispersive, due to the fast pace and large number of participants. In addition, it was not always easy to maintain the children’s attention, especially with the younger (<1year). Therefore, families proposed a reorganization of the video calls, decreasing the number of participants, slowing down the pace and reducing the free discussion in favor of activities with children as active learners. Following these suggestions, the Aeris team decided to diminish the number of children per call: from the 15 initial participants, some sub-groups were formed with approximately 10 children. This resulted in less chaotic meetings and gave the possibility to all children of interacting during the calls. These findings are in line with what reported by Szente (2020), who shared reflections on over 50 live Zoom instructional lessons with toddlers. In accordance with Aeris’ experience, children responded well to songs, engaging stories, dances, and rhythmic activities. However, when the online lessons lasted more than 20min, they seemed to be losing their interest. Finally, also Szente (2020) noticed that video calls tended to be chaotic if the participating children were more than 10–15.

In general, families found the activities proposed by the Aeris team engaging, well organized and useful for child development. Moreover, the Aeris team noticed an increased participation in the meetings during the pandemic than before. Indeed, the remote mode also allowed the more committed parents to attend the meetings, and this is the reason why the Aeris team decided to keep this participation option also after the COVID-19 outbreak. The same positive feeling was perceived in other worldwide realities, in which homeschool was well received by pupils and parents (Bubb and Jones, 2020; Garbe et al., 2020; Szente, 2020), or at least it was perceived as acceptable (Hafidz et al., 2020; Zhao et al., 2020). Despite these positive experiences, worldwide there is the common feeling that the e-learning needs to be improved (Fauzi and Sastra Khusuma, 2020; Putri et al., 2020; Rasmitadila et al., 2020), technology implemented (Dube, 2020; Fauzi and Sastra Khusuma, 2020; Rasmitadila et al., 2020), teachers more trained (Dias et al., 2020; Putri et al., 2020; Szente, 2020), and integrated grade-specific approaches are needed (Zhao et al., 2020). In general, teachers found a lower learning quality with respect to traditional lessons (Lucisano, 2020), and families had negative beliefs about the values and benefits of online learning, retaining that the family-school partnerships has not yielded compelling results and preferring traditional methods (Dong et al., 2020; Firmanto et al., 2020; Harper et al., 2021; Lee et al., 2021; Thorell et al., 2021). These findings differ from the Aeris reality, but we must point out that the initiatives described in this manuscript are specifically thought for 0–3 aged children, therefore dealing with simple activities that use materials easily available at home. When considering the previously mentioned studies the situation differs, since they deal with school age children (>3years old) who need more structured activities for their development, therefore more demanding to implement.

When it comes to children, either toddlers or school aged, what emerges is that the active participation of families is fundamental for obtaining convincing results during distance educational activities. Without the mutual collaboration between educators and families, it is impossible to hope for child cognitive and behavioral development. Several studies reported a high family engagement, demonstrating the intent of the caregivers to act as proactive actors in child development (Bubb and Jones, 2020; Hafidz et al., 2020; Kim, 2020; Novianti and Garzia, 2020; Rasmitadila et al., 2020; Sari and Maningtyas, 2020; Panaoura, 2021). This was found also in the Aeris reality, in which most of the families actively participated in the proposed activities. In contrast, teachers in other contexts had problems in collaborating with parents (Fauzi and Sastra Khusuma, 2020; Lucisano, 2020; Pek and Mee, 2020), while parents encountered problems with the e-learning due to their lack of time, professional knowledge in supporting children’s online learning (Dong et al., 2020; Garbe et al., 2020; Listyaningrum et al., 2020), and/or insufficient support from schools (Thorell et al., 2021). It is interesting to notice that studies in which parents were not sufficiently involved coincide with those previously mentioned for the unsuccess of the e-learning with respect the traditional methods, highlighting the importance of mutual collaboration between educational services and families (Phillips and Lowenstein, 2011; Bianco and Lecce, 2016). Despite the Aeris team was able to engage most of the families; the cooperative perceived that the pandemic had emphasized social differences among people. Indeed, not all of them participated in the distance initiatives, and the “left out-families” were usually those with fewer technical, cultural, and cognitive tools. However, Aeris lessened this phenomenon increasing communication especially with the most fragile and less responsive families, which usually were the foreign ones. Therefore, the emails were written in English, French, and Arabic, and some of the proposed videos were also in English other than Italian (i.e., the songs and nursery-rhymes). An improvement to reach more effectively these families might consist in proposing all the initiatives at least in double language (i.e., English and Italian). In addition, some families encountered organizational issues. Indeed, some of the parents had to assist more than one child during distance educational activities, while others never stopped working and, therefore, they were not able to connect to the online activities. Finally, some of the caregivers were grandparents who had no technological skills. A useful improvement in these situations may consist in making all the contents available in the cloud, to give the family the possibility of making them up when they have time.

To conclude, the role of parents is crucial in maintaining children’s well-being and education, especially during critical situations like this lockdown. It is essential to individuate parents’ needs and support them in addressing their educational role. However, this does not always happen, and the pandemic highlighted the need of mentoring families in parenting their children during learning from home (Listyaningrum et al., 2020; Meoded Karabanov et al., 2021). This pilot study could be a reference for policy-makers and managers of educational services in implementing activities and initiatives aimed at filling the gap between families and schools. Indeed, it contains suggestions for other Early Childhood Services in Italy or abroad, and it could be a useful tool for future organization of other services, since we are still facing short local school closure (UNESCO Website, 2021). Even if the results may be dependent on the social context and national environment, a similar model could be implemented after its adaptation to the local habits and behaviors.

Although this work can be a useful guide for future organization of educational services during homeschooling, it has some limitations. Firstly, it lacks of an assessment of children and parents’ wellbeing through validated instruments, and the measured variables are only parent-reported; indeed, the proposed survey evaluates families’ satisfaction about the organized activities, but not the effects of these initiatives on children development and parents’ stress levels. Moreover, due to the unpreparedness that characterized the beginning of the pandemic, the study lacks of a baseline evaluation of family wellbeing and needs (e.g., specific needs linked to the lockdown) before the starting of online activities. Finally, a comparison with early childhood services that acted in different ways is missing, which could be useful for identifying and implementing the best strategies in engaging families. Therefore, we will dedicate future works to the assessment of child development and children and parents’ wellbeing after the school closure using validated instruments, and to the evaluation of other actions/ways that other early childhood services implemented to generalize these findings.

## Data Availability Statement

The raw data supporting the conclusions of this article are openly available in Zenodo at DOI 10.5281/zenodo.5149218 (link: https://zenodo.org/record/5149219#.YYUD-LVKhPY, accessed on 30 July 2021).

## Ethics Statement

Ethical review and approval was not required for the study on human participants in accordance with the local legislation and institutional requirements. Written informed consent for participation was not required for this study in accordance with the national legislation and the institutional requirements.

## Author Contributions

RN: research design, data analysis and interpretation, drafting and revision of the article, and final approval of the version to be submitted. EB: research conception and design, data interpretation, revision of the article, and final approval of the version to be submitted. GC: research conception and design, instrument development, data collection, revision of the manuscript, and final approval of the version to be submitted. GG: research conception and design, instrument development, and data collection. LS: website development, data collection, revision of manuscript, and final approval of the version to be submitted. GR and CC: final approval of the version to be submitted. All authors contributed to the article and approved the submitted version.

## Funding

This study was supported by the Italian Ministry of Health (Ricerca Corrente 2021 to EB).

## Conflict of Interest

The authors declare that the research was conducted in the absence of any commercial or financial relationships that could be construed as a potential conflict of interest.

## Publisher’s Note

All claims expressed in this article are solely those of the authors and do not necessarily represent those of their affiliated organizations, or those of the publisher, the editors and the reviewers. Any product that may be evaluated in this article, or claim that may be made by its manufacturer, is not guaranteed or endorsed by the publisher.

## Footnotes

^1^ https://sites.google.com/coopaeris.it/smart-edu

^2^ https://www.facebook.com/aerisinfanzia

^3^ https://www.facebook.com/Spaziogioco.Papaveriepapere

^4^ https://www.facebook.com/smartedu.aeris
