# Supplementary material for: Engagement of Families Attending Early Childhood Services During 5-Month School Closure Due to COVID-19: An Italian Experience
Source: Front Psychol. 2021 Nov 18;12:722834. doi: 10.3389/fpsyg.2021.722834 (PMC8639320; doi:10.3389/fpsyg.2021.722834)
Supplement: Supplementary file 1 [file Table_1.DOCX]

1. Child’s age (in months): ________________________________________________________
2. Which childhood service does the child attend?

- Agrate nursery
- Cambiago nursery
- Robbiate nursery
- Vaprio nursery
- Agrate Spazio Giochi
- Trezzo Spazio Giochi
- Vaprio Spazio Giochi
- Cambiago Spazio Giochi
- Agrate Merenda in Gioco
- Agrate Servizio Ponte

1. Does your family have a device to participate in online activities?

- Yes
- No

1. If yes, which one?_____________________________________________________________
2. Does the device have an internet connection?

- Yes
- No

1. If yes, which are its characteristics?

- Internet without limits
- With limits/GIGA consumption

1. If not, which are the difficulties/limitations? ________________________________________
2. Do you have another child/any other children in your family?
   - Yes
   - No
3. If yes, how many they are?______________________________________________________
4. If yes, how old are they? ________________________________________________________
5. If yes, do the other children use smartphone, tablets or computers to attend online activities?___________________________________________________________________
6. During the day or the week, do you participate in the creative proposals suggested by the educators?
   - Yes
   - No
7. Is your child enthusiastic when you ask him/her to participate in the activities? (5-point Likert scale, 1: not at all, 5: a lot):
   - 1
   - 2
   - 3
   - 4
   - 5
8. During the day, does the child ask to participate in the educators’ proposals (e.g. songs, reading, stories…)? (5-point Likert scale, 1: not at all, 5: a lot):
   - 1
   - 2
   - 3
   - 4
   - 5
9. How engaging and appropriate do you find the proposed activities? (5-point Likert scale, 1: not at all, 5: a lot):
   - 1
   - 2
   - 3
   - 4
   - 5
10. Is your child actively engaged by the proposed activities? (5-point Likert scale, 1: not at all, 5: a lot):
    - 1
    - 2
    - 3
    - 4
    - 5
11. Do you feel engaged and do you participate in the proposed activities? (5-point Likert scale, 1: not at all, 5: a lot):
    - 1
    - 2
    - 3
    - 4
    - 5
12. Do you have any suggestions about the activities dedicated to the children?_______________
13. Do you have any suggestions about the communication methods and tools used during these activities?___________________________________________________________________
14. Did you watch the shared videos dealing with child growth?
    - Yes
    - No
15. Did you find these videos useful? (5-point Likert scale, 1: not at all, 5: a lot):
    - 1
    - 2
    - 3
    - 4
    - 5
16. Did you read the shared documents dealing with child growth?
    - Yes
    - No
17. Did you find these documents useful? (5-point Likert scale, 1: not at all, 5: a lot):
    - 1
    - 2
    - 3
    - 4
    - 5
18. Did you participate in individual calls/videocalls with the educators?
    - Yes
    - No
19. Did you find these individual calls with the educators useful? (5-point Likert scale, 1: not at all, 5: a lot):
    - 1
    - 2
    - 3
    - 4
    - 5
20. Did you participate in individual calls/videocalls with the pedagogist?
    - Yes
    - No
21. Did you find these individual calls with the pedagogist useful? (5-point Likert scale, 1: not at all, 5: a lot):
    - 1
    - 2
    - 3
    - 4
    - 5
22. Did you participate in group videocalls with the educators and other families?
    - Yes
    - No
23. Did you find these group videocalls useful? (5-point Likert scale, 1: not at all, 5: a lot):
    - 1
    - 2
    - 3
    - 4
    - 5
24. Did you use the Smart-EDU website?
    - Yes
    - No
25. Did you find the Smart-EDU website useful? (5-point Likert scale, 1: not at all, 5: a lot):
    - 1
    - 2
    - 3
    - 4
    - 5
26. Did you use the WhatsApp groups/broadcasts?
    - Yes
    - No
27. Did you find the WhatsApp groups/broadcasts useful? (5-point Likert scale, 1: not at all, 5: a lot):
    - 1
    - 2
    - 3
    - 4
    - 5
28. Do you find useful to have updates about the activities and what other families do as well?
    - Yes
    - No
29. If yes, using which media (e.g. WhatsApp, emails…)?__________________________________
30. Does the child pay attention during the videocalls? (5-point Likert scale, 1: not at all, 5: a lot):
    - 1
    - 2
    - 3
    - 4
    - 5
31. Does the child actively participate in the activities during the videocalls? (5-point Likert scale, 1: not at all, 5: a lot):
    - 1
    - 2
    - 3
    - 4
    - 5
32. Is eye and verbal contact with the other families important? (5-point Likert scale, 1: not at all, 5: a lot):
    - 1
    - 2
    - 3
    - 4
    - 5
33. Is the duration of the videocalls suitable for a child? (5-point Likert scale, 1: not at all, 5: a lot):
    - 1
    - 2
    - 3
    - 4
    - 5
34. Do you feel engaged to perform the proposed activities? (5-point Likert scale, 1: not at all, 5: a lot):
    - 1
    - 2
    - 3
    - 4
    - 5
35. Do you find interesting and useful online group meetings organized exclusively for parents? (5-point Likert scale, 1: not at all, 5: a lot):
    - 1
    - 2
    - 3
    - 4
    - 5
36. Do you have any suggestions for improving the videocalls?_____________________________
